# Surgical Outcomes of Lower Genitourinary Fistula Repair: A Prospective Cohort Study From an Indian Tertiary Hospital

**DOI:** 10.7759/cureus.97910

**Published:** 2025-11-27

**Authors:** Mughakato H Sema, Vivek Singh, Mohd. Rehan Akhtar, Jitender Yadav, Sumit Pal, Mukul K Singh, Vishwajeet Singh

**Affiliations:** 1 Urology, King George's Medical University, Lucknow, IND

**Keywords:** laparoscopy, ureterovaginal fistula, urethrovaginal fistula, vesicouterine fistula, vesicovaginal fistula

## Abstract

Introduction: Lower genitourinary tract fistulas remain a debilitating condition causing continuous urinary leakage, with vesicovaginal fistula (VVF) being the most common subtype. Despite advances in minimally invasive techniques, the optimal surgical approach remains debated. This study aimed to evaluate and compare the short-term surgical outcomes of laparoscopic, open transabdominal, and transvaginal fistula repairs, while assessing the influence of fistula characteristics, recurrence status, and interposition techniques on success rates.

Materials and methods: In this prospective study, 104 women underwent surgical repair: VVF (n=81, 77.8%), ureterovaginal fistula (UVF; n=15, 14.4%), vesicouterine fistula (VUF; n=4, 3.8%), and urethrovaginal fistula (UVVF; n=4, 3.8%). Preoperative evaluation included cystoscopy, vaginoscopy, and selective imaging. Surgical approaches were tailored by fistula site, size, and complexity. Interposition techniques (omental flap, Martius flap, or Surgicel scaffold) and adjunctive procedures (ureteric reimplantation, augmentation cystoplasty, Boari flap, and psoas hitch) were used as indicated. The primary outcome was fistula closure at three months.

Results: At three-month follow-up, the overall success rate was 97 (93.3%). Among VVFs, closure rates were comparable: laparoscopic (n=18, 90%), open (n=32, 94.1%), and transvaginal (n=25, 92.6%) (p=0.856). All UVF (n=15, 100%), VUF (n=4, 100%), and UVVF (n=4, 100%) cases were successfully treated. Perioperative outcomes favored the transvaginal route for the shortest operative time, while laparoscopy reduced blood loss and hospital stay compared to open repair. Postoperative complications (Clavien-Dindo ≥II) occurred only in the VVF group (n=10, 12.3%).

Conclusions: Surgical repair of lower genitourinary fistulas achieved high short-term success across laparoscopic, open, and transvaginal approaches. Success rates were comparable, while perioperative profiles differed: transvaginal repair minimized operative time, laparoscopy reduced blood loss and hospital stay, and open repair was advantageous for complex cases. Tailoring the surgical approach to fistula characteristics ensures optimal outcomes.

## Introduction

Lower genitourinary tract fistulas represent a devastating clinical condition, characterized by abnormal communication between the urinary and genital tracts. These fistulas lead to continuous urinary leakage, perineal skin excoriation, recurrent urinary tract infections, infertility, dyspareunia, and severe psychosocial consequences [1]. For affected females, the condition is not merely a surgical disease but a profound social and psychological burden, often resulting in stigma, marital disruption, and impaired quality of life. Despite being preventable in most instances, the global burden of female genitourinary fistulas remains significant [2]. The World Health Organization estimates that more than two million women, predominantly in sub-Saharan Africa and South Asia, currently live with untreated fistulas. In these regions, obstructed or prolonged labor remains the principal etiology, reflecting inequities in maternal healthcare delivery [3]. In contrast, in high-income countries, iatrogenic injuries related to gynecologic or obstetric procedures, particularly hysterectomy and cesarean section, constitute the predominant cause. This dichotomy highlights the importance of regional context in understanding disease etiology and tailoring management strategies [4].

The majority of fistulas involve the bladder and vagina (vesicovaginal fistula (VVF)), followed by ureterovaginal fistula (UVF), vesicouterine fistula (VUF), and urethrovaginal fistula (UVVF). Each subtype presents unique clinical challenges [5]. VVFs are most common and vary widely in location, size, and complexity, dictating individualized repair strategies. UVFs often present with preserved continence but continuous vaginal leakage, requiring early recognition and timely repair to preserve renal function. VUFs, typically associated with cesarean delivery, manifest as menouria (cyclical hematuria during menstruation) or secondary infertility and require meticulous surgical dissection for successful correction [6]. UVVFs represent one of the most complex scenarios, as involvement of the continence mechanism or bladder neck can compromise both anatomic closure and long-term urinary control.

Surgical repair remains the cornerstone of fistula management. The transvaginal route is widely accepted as the preferred approach for accessible fistulas, especially those that are low-lying, small in size (<2.5 cm), or located at the trigone or bladder neck. Its advantages include minimal invasiveness, shorter operative times, reduced blood loss, lower postoperative morbidity, and faster recovery [7]. However, limitations include restricted exposure in large, high-supratrigonal, or recurrent fistulas, or when concomitant pathology requires abdominal access. In such cases, open transabdominal repair provides superior exposure, allows for excision of scar tissue, facilitates use of an omental flap for interposition, and permits simultaneous ancillary procedures such as augmentation cystoplasty or ureteric reimplantation [8,9]. Laparoscopic repair, and more recently robotic surgery, have emerged as minimally invasive alternatives to open surgery, particularly for supratrigonal fistulas. These approaches replicate the principles of abdominal repair while offering reduced morbidity, shorter hospitalization, and faster convalescence, though at the expense of longer operative times and the need for specialized expertise [10].

A critical adjunct in complex fistula surgery is the use of tissue interposition flaps or grafts. The omentum, Martius labial fat pad, and synthetic scaffolds (such as oxidized cellulose and gelatine sponge) are employed to reinforce closure, provide a vascularized layer, and minimize recurrence [11]. Their role, however, remains debated. While many surgeons advocate their use in recurrent or large defects, others report equivalent outcomes with primary layered closure alone when tissues are healthy and tension-free. Furthermore, ancillary reconstructive procedures such as ureteric reimplantation, Boari flap, psoas hitch, and augmentation cystoplasty may be required in select cases where the fistula involves the ureteric orifices, compromises bladder capacity, or coexists with additional pelvic pathology [12]. These factors underscore the complexity and heterogeneity of fistula management, emphasizing the need for individualized surgical planning.

Despite advances in surgical techniques, there is no universal consensus on the optimal route or method of repair. Most available studies are retrospective, limited to single institutions, and focus on one technique [13]. Comparative prospective data evaluating laparoscopic, open, and vaginal approaches in the same cohort are limited, particularly from low- and middle-income country settings where the etiological spectrum is broader and resource availability is variable. This gap limits the ability to formulate evidence-based recommendations for surgical decision-making in diverse clinical environments [14].

The present study was therefore undertaken to evaluate the surgical outcomes of lower genitourinary tract fistula repairs in a prospective cohort. Specifically, we aimed to compare laparoscopic, open transabdominal, and transvaginal techniques with respect to surgical success, perioperative parameters (operative time, blood loss, hospital stay), and complication profile using the Clavien-Dindo classification. We also analyzed the influence of fistula characteristics, recurrence status, duration of incontinence, interposition techniques, and the need for ancillary reconstructive procedures on surgical success.

## Materials and methods

Study design

This was a prospective study conducted in the Department of Urology, King George’s Medical University, Lucknow, India, between April 2023 and January 2025. A total of 104 consecutive females diagnosed with lower genitourinary tract fistulas were enrolled for surgical repair. Inclusion criteria include patients presenting with lower genitourinary tract fistulas requiring surgical repair. Diagnosis was confirmed by clinical evaluation, cystoscopy, and vaginoscopy, with additional imaging, such as CT urography, intravenous urography, or retrograde pyelography, performed when indicated. Only patients with adequate clinical documentation, including obstetric and gynecologic history and physical examination findings, were included. Exclusion criteria include fistulas secondary to pelvic malignancy or prior pelvic radiotherapy; fistulas of inflammatory origin, such as tuberculosis or Crohn’s disease (to ensure homogeneity of outcomes); pregnancy at the time of evaluation (to avoid perioperative risk and confounding obstetric factors); incomplete clinical records; or refusal to undergo surgical repair or provide informed consent. All patients were followed for three months postoperatively to assess surgical success, ensuring uniform outcome measurement across the cohort.

The study protocol was approved by the Institutional Ethics Committee of King George's Medical University (approval number: XXV-PGTSC-IIA/P53) and was conducted in accordance with the principles of the Declaration of Helsinki. Written informed consent was obtained from all participants.

Preoperative evaluation and workup

All patients underwent a standardized preoperative assessment that included cystoscopy and vaginoscopy to understand the site, size, and number of fistulas, evaluate vaginal mucosal condition, and determine the adequacy of the vaginal lumen. Cystoscopy further assessed the position and patency of bilateral ureteric orifices in relation to fistula margins. In patients with UVFs, retrograde ureteropyelography was performed; in those with associated strictures, percutaneous nephrostomy with antegrade contrast studies was used to delineate the strictures. Vaginal examination assessed cervical status, vaginal vault integrity, and other relevant pelvic findings. Baseline laboratory investigations included blood chemistry, urinalysis, and urine culture to evaluate systemic status and exclude urinary tract infection. Ultrasound of the kidneys, ureters, and bladder (USG-KUB) was performed in all cases to assess upper tract function and bladder morphology. Additional imaging was selectively applied based on clinical indication: CT urography for complex anatomy or ureteric involvement.

Surgical techniques

The surgical approach was tailored to the fistula site, size, complexity, and vaginal accessibility. Transvaginal repair was undertaken for low-lying or trigonal VVFs with an adequate vaginal lumen and healthy tissue, including UVVFs and VVFs with associated rectovaginal fistulas. Open transabdominal repair was selected for supratrigonal or large trigonal fistulas, cases with a narrow or scarred vaginal canal, or when concomitant pelvic pathology, reduced bladder capacity requiring augmentation, or simultaneous rectovaginal fistula repair was present. Laparoscopic repair was reserved for small (<2.5 cm) supratrigonal fistulas in which minimally invasive closure could provide outcomes comparable to open surgery with reduced morbidity; the final decision between vaginal and laparoscopic repair depended on fistula size, location, surgeon expertise, and patient preference. UVFs were managed with ureteric reimplantation via the laparoscopic route in unilateral cases with favorable anatomy and open repair for bilateral disease, extensive scarring, or long strictures requiring adjunctive procedures such as a psoas hitch or a Boari flap. Interposition flaps (omentum, Martius, Surgicel scaffold, or gelatin sponge) were selectively employed to reinforce the repair in cases with large or recurrent fistulas, poor tissue quality, or surgeon preference. While patients were not randomized, introducing potential selection bias, the approach reflects pragmatic decision-making in a high-volume tertiary referral setting.

Transvaginal Repair of Vesicovaginal Fistulas (n=27)

Cystoscopy was performed in all cases, with ureteric stenting in those where the orifices were close to the fistula. A traction catheter was passed through the fistula tract to facilitate exposure. A circumferential incision was made ~5 mm from the fistula margin, followed by sharp dissection to separate the bladder and vaginal walls. The bladder was closed in a watertight manner with interrupted 3-0 polyglactin sutures, and the vagina was closed in one layer. Interposition was performed using a Martius flap in 16 cases and a Surgicel scaffold in 11 cases. Per-urethral drainage was routine, with suprapubic drainage added in complex cases. Catheters were removed after cystographic confirmation at three to four weeks, or later if extravasation persisted.

Open Transabdominal Vesicovaginal Fistula Repair (Modified O’Connor; n=34)

Through a Pfannenstiel incision in 12 patients and an infra-umbilical midline incision in 22 patients, the bladder was exposed, and a posterior cystotomy was made. Small fistula tracts (n=14) were encircled entirely and excised, while large defects >2.5 cm (n=20) were managed with a rotational bladder flap to achieve tension-free closure. Vaginal closure was performed with interrupted 3-0 polyglactin sutures, followed by bladder closure in a single continuous layer. An omental flap was interposed in all 34 cases to reinforce the repair. Adjunctive procedures included augmentation ileo-cystoplasty in two patients and ureteric reimplantation in four patients. Both per-urethral and suprapubic catheters were placed, with removal after cystographic confirmation at three to four weeks.

Laparoscopic Vesicovaginal Fistula Repair (n=20)

All patients underwent preoperative ureteric stenting. A 10 mm camera port and two 5 mm working ports were placed in a steep Trendelenburg position. The fistula was approached extravesically in nine cases and transvesically in 11 cases. The vaginal defect was closed with interrupted 3-0 polyglactin sutures, followed by a two-layer bladder closure using 3-0 polyglactin sutures. An omental flap was interposed in all cases. Bladder integrity was confirmed with a methylene-blue leak test, and pelvic drains were removed when output decreased to <30 mL/24 hours.

Ureterovaginal Fistula Repair (n=13)

Urinary diversion was achieved preoperatively with percutaneous nephrostomy or double-J stenting as indicated. Definitive repair was performed using the extravesical Lich-Gregoir ureteric reimplantation technique. Seven patients underwent open repair (including four bilateral and three unilateral), while six underwent laparoscopic repair (all unilateral). The ureter was mobilized and spatulated before being anastomosed to the bladder mucosa over a 4.8-6F stent, and detrusor flaps were closed to create a submucosal tunnel. A psoas hitch or Boari flap was added when long ureteric strictures were present. Stents were removed after four to six weeks.

Vesicouterine Fistula Repair (n=10)

VUFs were managed laparoscopically (n=2) and open (n=2). Cystoscopy guidance aided localization. After sharp dissection, the uterine and bladder defects were closed in layers, and an omental flap was routinely interposed. Catheters were removed after cystographic confirmation at three to four weeks.

Postoperative care and follow-up

Immediate Postoperative Management

All patients received standardized postoperative care, which included antibiotics for five to seven days, as per routine urine culture. A per-urethral Foley catheter was placed in all patients, and a suprapubic catheter was added selectively during complex or open repairs. Pelvic drains were routinely used during open and laparoscopic abdominal repairs and were removed when output fell below 30 mL over 24 hours. Patients were told to abstain from sexual intercourse for three months.

Catheter and Stent Management

Bladder drainage was maintained for three to four weeks. Before catheter removal, all patients underwent a cystogram to confirm bladder integrity. In nine patients with persistent extravasation, catheterization was prolonged for an additional one to two weeks until complete healing was demonstrated. For UVF repairs, ureteric stents were retained for four to six weeks and removed under cystoscopic guidance.

Three-Month Follow-Up

All patients were evaluated at a uniform follow-up visit three months after surgery. Clinical assessment included continence status, wound healing, and symptom review. Investigations performed at this visit included a USG-KUB with post-void residual to evaluate the upper tract and a urinalysis to exclude infection. Uroflowmetry was performed selectively in symptomatic patients.

Outcome Assessment

Surgical success was defined as complete restoration of the anatomical defect with restoration of vaginal and bladder function at the three-month follow-up. Postoperative complications were recorded prospectively from inpatient charts and outpatient visits and were graded according to the Clavien-Dindo classification.

Statistical analysis

Data were entered into a predesigned pro forma and analyzed using SPSS Statistics version 24.0 (IBM Corp. Released 2016. IBM SPSS Statistics for Windows, Version 24.0. Armonk, NY: IBM Corp.). Continuous variables such as operative time, intraoperative blood loss, and hospital stay were expressed as mean ± standard deviation (SD) and compared across groups using one-way analysis of variance (ANOVA), followed by post hoc Tukey’s test where appropriate [15]. Categorical variables, including surgical success rates, recurrence, complication profile (Clavien-Dindo classification), and the use of interposition techniques, were expressed as numbers and percentages and compared using the chi-square test or Fisher’s exact test as applicable. Subgroup analyses were performed for VVF repairs to compare transvaginal, open transabdominal, and laparoscopic approaches, as these represented the largest cohort with all three modalities. For UVF, VUF, and UVVF, outcomes were presented descriptively due to smaller sample sizes. A p-value <0.05 was considered statistically significant.

## Results

A total of 104 females with lower genitourinary fistulas were included VVF (n=81, 77.8%), UVF (n=15, 14.4%), UVVF (n=4, 3.8%), and VU (n=4, 3.8%). The mean age of the cohort was 34.8 ± 9.1 years (range: 6-71), with UVF patients being the oldest subgroup (37.9 ± 7.6 years, 28-48) and UVVF patients the youngest (24.5 ± 8.4 years, corrected range: 18-35). The median preoperative duration of incontinence was six months (IQR: 5-12), the longest among UVVF patients (14 months, IQR: 9-22). Most fistulas were supratrigonal (n=62, 59.6%) and <2.5 cm in size (n=82, 78.8%), with primary cases accounting for 90 (86.5%). The leading etiology was total abdominal hysterectomy (n=67, 64.4%), followed by obstructed labor (n=18, 17.3%) and lower-segment cesarean section (n=9, 8.6%) (Table 1).

**Table 1:** Baseline demographics, fistula characteristics, and etiology of lower genitourinary tract fistulas Percentages are calculated within each subgroup unless otherwise stated. Recurrent is defined as a prior failed fistula repair. IQR: interquartile range, SD: standard deviation, UVF: ureterovaginal fistula, UVVF: urethrovaginal fistula, VUF: vesicouterine fistula, VVF: vesicovaginal fistula, LSCS: lower-segment cesarean section

Parameter	UVF (n=15)	UVVF (n=4)	VUF (n=4)	VVF (n=81)	Total (n=104)
Demographics					
Age (years), mean ± SD (range)	37.9 ± 7.6 (28-48)	24.5 ± 8.4 (23-56)	32.5 ± 4.6 (26-36)	35.2 ± 9.4 (18-71)	34.8 ± 9.1 (6-71)
Duration of incontinence (months), median (IQR)	4 ( 3-5)	14 (9-22)	9 (5-12)	6 (5-12)	6 (5-12)
Fistula characteristics					
Site – supratrigonal	0	0	4 (100%)	58(71.6%)	62 (59.6%)
Site – trigonal/bladder neck	0	4 (100%)	0	23 (28.4%)	27 (25.9%)
Size <2.5 cm	12 (80.0%)	2 (50.0%)	3 (75.0%)	65 (80.2%)	82 (78.8%)
Size ≥2.5 cm	3 (20.0%)	2 (50.0%)	1 (25.0%)	16 (19.8%)	22 (21.2%)
Primary cases	14 (93.3%)	2 (50.0%)	4 (100%)	70 (86.4%)	90 (86.5%)
Recurrent cases	1 (6.7%)	2 (50.0%)	0	11 (13.6%)	14 (13.5%)
Etiology					
Total abdominal hysterectomy	14 (93.3%)	0	0	53 (65.4%)	67 (64.4%)
LSCS	1 (6.7%)	0	3 (75.0%)	5 (6.2%)	9 (8.6%)
LSCS + Copper-T	0	0	1 (25.0%)	0	1 (1.0%)
Obstructed labor	0	2 (50.0%)	0	16 (19.8%)	18 (17.3%)
Road traffic accident	0	2 (50.0%)	0	2 (2.5%)	4 (3.8%)
Transvaginal hysterectomy	0	0	0	3 (3.7%)	3 (2.9%)
Dilatation and curettage	0	0	0	1 (1.2%)	1 (1.0%)
Foreign body removal	0	0	0	1(1.2%)	1 (1.0%)

Distribution of fistula types, surgical approaches, and adjunctive procedures

In the VVF cohort (n=81), laparoscopic repair was performed in 20 patients (24.7%), open transabdominal repair in 34 patients (42.0%), and transvaginal repair in 27 patients (33.3%). Omental flap interposition was used in all laparoscopic (20/20) and open abdominal cases (34/34), while transvaginal repairs employed Martius flap interposition in 16/27 (59.3%) and Surgicel scaffold/gelatine sponge reinforcement in 11/27 (40.7%).

Adjunctive procedures in the open group included augmentation ileo-cystoplasty (2/34, 5.9%) and ureteric reimplantation (4/34, 11.8%). Among UVF cases (n=15), laparoscopic ureteric reimplantation was undertaken in six patients (40.0%), and open reimplantation in seven patients (46.7%). Adjunctive procedures were performed exclusively in the open reimplantation group, including Boari flap reimplantation (1/7, 14.3%), psoas hitch (2/7, 28.6%), and bilateral ureteric reimplantation (2/7, 28.6%). Conservative double-J stenting alone was successful in 2/15 patients (13.3%). In the VUF group (n=4), repair was evenly distributed between laparoscopic (2/4, 50.0%) and open transabdominal (2/4, 50.0%) approaches, with omental flap interposition used in all cases (4/4, 100%). In the UVVF group (n=4), two patients (50.0%) underwent multilayered transvaginal repair with Martius flap interposition. In comparison, two patients (50.0%) with extensive bladder neck involvement required bladder neck closure reinforced with a Martius flap and creation of continent catheterizable channels (Mitrofanoff in one, Monti in one) (Tables 2-3).

**Table 2:** Distribution of fistula types and corresponding surgical or conservative management approaches UVF: ureterovaginal fistula, UVVF: urethrovaginal fistula, VUF: vesicouterine fistula, VVF: vesicovaginal fistula

Fistula type	n	(%)	Surgical/conservative approach	n	(%)
VVF	81	77.9	Laparoscopic repair	20	24.7
			Open transabdominal repair	34	42
						Transvaginal repair	16	19.7
			11	13.5		
			UVF	15	14.4	Laparoscopic ureteric reimplantation	6	40
Open ureteric reimplantation	7	46.7			
						Conservative management (double-J stenting)	2	13.3
						VUF	4	3.9	Laparoscopic repair	2	50
Open transabdominal repair	2	50						
UVVF	4	3.9	Transvaginal multilayered repair	2	50
			Bladder neck closure + continent catheterizable channel	2	50

**Table 3:** Distribution of patients undergoing various open ureteric reimplantation and adjunctive reconstructive procedures

Interposition techniques	Adjunctive procedures	N	%
Omental flap	Augmentation cystoplasty	2	6.25
	Ureteric reimplantation	4	12.5
Open ureteric reimplantation	Boari flap	1	14.2
	bilateral Psoas hitch	2	28.57
	Reimplantation	2	28.57
Martius flap reinforcement	Mitrofanoff	1	50
	Monti	1	50

Perioperative outcomes

Among VVF repairs, perioperative outcomes varied significantly by surgical approach (Table 3). Transvaginal repair achieved the shortest mean operative time (86.7 ± 24.8 minutes), followed by open transabdominal (116.0 ± 27.1 minutes), while laparoscopic repair took the longest (140.1 ± 19.4 minutes; p<0.001). Intraoperative blood loss was significantly greater in open abdominal repairs (124.2 ± 29.7 mL) compared with both laparoscopic (94.7 ± 21.8 mL) and transvaginal (95.1 ± 28.3 mL) approaches (p = 0.001). Hospital stay was also longest after open abdominal repair (6.2 ± 2.0 days) compared to laparoscopic (4.8 ± 1.3 days) and transvaginal (5.0 ± 1.9 days) approaches (p=0.001). No perioperative blood transfusions were required in any group (Table 4).

**Table 4:** Comparison of perioperative outcomes (operative time, blood loss, and hospital stay) across laparoscopic, open transabdominal, and transvaginal VVF repairs SD: standard deviation, VVF: vesicovaginal fistula

Variable	Laparoscopic repair (n=20)	Open transabdominal repair (n=34)	Transvaginal repair (n=27)	Chi-square (p-value)
Operative time (min), mean ± SD	140.1 ± 19.4	116.0 ± 27.1	86.7 ± 24.8	<0.001
Blood loss (mL), mean ± SD	94.7 ± 21.8	124.2 ± 29.7	95.1 ± 28.3	0.001
Hospital stay (days), mean ± SD	4.8 ± 1.3	6.2 ± 2.0	5.0 ± 1.9	0.001

Postoperative outcomes

At three-month follow-up, the overall primary success rate across all fistula types was 96.2 (100/104), with resolution of urinary incontinence and no evidence of recurrence or persistent leakage. Among females with VVF (n=81), surgical closure was achieved in 74 patients (91.4%), while seven (8.6%) experienced persistent fistula requiring re-intervention. Success rates were comparable across surgical approaches: 90% (18/20) for laparoscopic repair, 94.1% (32/34) for open transabdominal repair, and 92.6% (25/27) for transvaginal repair (p=0.856).

All UVF patients (n=15) were ultimately cured, with 13 undergoing definitive surgical repair (six laparoscopic, seven open) and two managed successfully with double-J stenting. One patient had a persistent per-vaginal leak three months after stenting, required surgical reimplantation, and was subsequently cured, ensuring a 100% patient-level success rate. VUF (n=4) and UVVF (n=4) repairs also achieved 100% closure rates, regardless of surgical technique. Grade II and above complications were observed exclusively in the VVF cohort (10/81, 12.3%), distributed as Grade II (n=3) and Grade III (n=7) events. Grade II complications included urinary tract infection (n=2) and Martius flap wound dehiscence (n=1), while Grade III events were due to persistent fistulas necessitating secondary surgical intervention. No Grade ≥2 complications occurred in the UVF, VUF, or UVVF groups, and there was no mortality (Tables 5-6).

**Table 5:** Distribution of fistula types and corresponding surgical or conservative approaches UVF: ureterovaginal fistula, UVVF: urethrovaginal fistula, VUF: vesicouterine fistula, VVF: vesicovaginal fistula

Fistula type	n	(%)	Surgical/conservative approach	n	(%)
VVF	81	77.9	Laparoscopic repair	20	24.6
			Open transabdominal repair	34	41.9
			Transvaginal repair	27	33.3
UVF	15	14.4	Laparoscopic ureteric reimplantation	6	40.0
			Open ureteric reimplantation	7	46.7
			Conservative (DJ stenting)	2	13.3
VUF	4	3.9	Laparoscopic repair	2	50.0
			Open repair	2	50.0
UVVF	4	3.9	Transvaginal multilayer repair	2	50.0
			Bladder neck closure + catheterizable channel	2	50.0

**Table 6:** Outcomes and complications following surgical or conservative management of genitourinary fistulae UVF: ureterovaginal fistula, UVVF: urethrovaginal fistula, VUF: vesicouterine fistula, VVF: vesicovaginal fistula

Fistula type/procedure	Success (n)	(%)	Recurrence/persistence (n)	(%)	Complications (Clavien–Dindo)
VVF					
Laparoscopic repair	19	95.0	1	5.0	Grade II: 3; Grade III: 7
Open transabdominal repair	32	94.1	2	5.9	None
Transvaginal repair	26	96.3	1	3.7	None
UVF					
Laparoscopic ureteric reimplantation	6	100	0	0	None
Open ureteric reimplantation	7	100	0	0	None
Double-J stenting (conservative)	2	66.7	1	33.3	None
VUF	4	100	0	0	None
UVVF	4	100	0	0	None

Comparative analysis of surgical techniques

Among the 81 females undergoing VVF repair, outcomes were broadly comparable across transvaginal, open transabdominal, and laparoscopic approaches. The overall surgical success rate was high, with no significant difference between techniques (96.3% for transvaginal, 94.1% for open, and 95% for laparoscopic repair; p=0.927). Perioperative parameters varied significantly: open transabdominal repair was associated with the most significant blood loss (124.18 ± 29.68 mL), compared with laparoscopic (94.67 ± 21.81 mL) and transvaginal (95.13 ± 28.3 mL) approaches (p=0.001). Operative time was shortest in the transvaginal group (86.65 ± 24.84 minutes) and longest for laparoscopic repair (140.05 ± 19.35 minutes), with open repair intermediate at 116.0 ± 27.14 minutes (p<0.0001). Hospital stay was significantly longer after open repair (6.19 ± 1.99 days) than after laparoscopic (4.82 ± 1.33 days) or transvaginal (5.01 ± 1.91 days) approaches (p = 0.001). The complication profile did not differ significantly among groups, with Clavien-Dindo grade ≥2 events observed in 11.1% of transvaginal, 11.8% of open, and 15.0% of laparoscopic repairs (p=0.923). Interposition techniques were employed in all cases: omental flaps were universally used in open and laparoscopic repairs, while transvaginal repairs utilized either Martius flaps (n=16, success 93.8%) or Surgicel scaffolds/gelatine sponges (n=11, success 100%). Adjunctive procedures included augmentation cystoplasty (n=2) and ureteric reimplantation (n=4) in the open group, all of which had successful outcomes (Tables 7-9).

**Table 7:** Comparison of success rates and Clavien-Dindo ≥2 complications among patients undergoing transvaginal, open transabdominal, and laparoscopic VVF repairs VVF: vesicovaginal fistula

Parameter	Transvaginal repair (n=27)		Open transabdominal repair (n=34)		Laparoscopic repair (n=20)		Chi-square (p-value)
	n	%	n	%	n	%	
Success rate	26	96.3	32	94.1	19	95	0.927
Clavien-Dindo ≥2 complications	3	11.1	4	11.8	3	15	0.923

**Table 8:** Comparative perioperative and postoperative outcomes of VVF repair across transvaginal, open transabdominal, and laparoscopic approaches SD: standard deviation, VVF: vesicovaginal fistula

Parameter	Transvaginal repair (n=27)	Open transabdominal repair (n=34)	Laparoscopic repair (n=20)	Chi-square (p-value)
Mean blood loss (mL ± SD)	95.13 ± 28.3	124.18 ± 29.68	94.67 ± 21.81	0.001
Operative time (min ± SD)	86.65 ± 24.84	116.0 ± 27.14	140.05 ± 19.35	0.0001
Hospital stay (days ± SD)	5.01 ± 1.91	6.19 ± 1.99	4.82 ± 1.33	0.001

**Table 9:** Success rates of different interposition techniques used in fistula repair

Interposition technique	Sample size (n)	Success rate (%)
Martius flap	16	93.8%
Surgicel scaffold/gelatine sponge	11	100%
Omental flap	34	94.1%
Omental flap	20	95%

## Discussion

In this prospective cohort of 104 females with lower genitourinary fistulas, the overall three-month surgical success rate was 96.15%, defined as restoration of the anatomical defect and vaginal and bladder function at three-month follow-up. These short-term outcomes are consistent with contemporary series from specialized centers reporting 85-100% success in non-irradiated cohorts [16]. High success in our study likely reflects exclusion of radiotherapy-related fistulas, which are well known to heal less reliably (often <70%) and recur more frequently due to compromised tissue vascularity [17]. By focusing on iatrogenic/obstetric etiologies predominantly post-hysterectomy, our results reflect conditions favorable for primary healing when meticulous surgical principles are applied [18].

Among VVF repairs, success did not differ by route (transvaginal 96.3%, open 94.1%, laparoscopic 95.0%; p=0.927), aligning with meta-analytic data showing no intrinsic advantage of abdominal versus vaginal access when cases are appropriately selected [18,19]. Each approach, however, displayed distinct perioperative profiles. In our series, transvaginal repair had the shortest operative time and the least blood loss; this is consistent with the literature, which favors the vaginal route for accessible, low- or mid-vaginal fistulas due to reduced operative trauma and recovery time with equivalent efficacy [20]. Open transabdominal repair resulted in greater blood loss and a longer hospital stay, yet enabled management of complex scenarios (large supratrigonal defects, concurrent pelvic pathology) with high success, consistent with prior recommendations for high-lying or complicated fistulas, particularly when omentum can be interposed [21]. Laparoscopic repair matched open and vaginal success while reducing blood loss and length of stay relative to open, at the cost of longer operative time, findings in line with systematic reviews showing minimally invasive repairs achieve comparable closure with lower morbidity [22].

Interposition was used judiciously. All abdominal repairs received omental flaps; transvaginal repairs used Martius flaps (59%) or an oxidized regenerated cellulose scaffold/gelatine sponge (41%). In our transvaginal subset, success was 100% with the surgical scaffold/gelatine sponge (11/11) and 93.8% with Martius (14/16), though the numbers were small and not significantly different. Evidence syntheses suggest routine Martius interposition does not significantly increase primary closure rates when a tension-free, watertight repair is achieved in healthy tissue [23,24]. Similarly, omental/peritoneal interposition has not consistently shown independent improvement in primary success across all-comer cohorts [25]. Nevertheless, expert consensus supports the use of flaps in recurrent, large, ischemic, or irradiated defects to augment healing; our exclusive use in complex cases likely contributed to the uniformly high ultimate cure [26].

Fistula characteristics guided route selection. Low-lying (<2-2.5 cm) defects with adequate vaginal access were preferably repaired transvaginally; large (≥2.5-3 cm), high/trigonal, recurrent, or ureteric-orifice-adjacent defects more often required an abdominal route (open or laparoscopic), frequently with interposition or ancillary reconstruction, an approach consistent with commonly used “simple/complex” classifications [27]. Although failures in our cohort occurred only among VVF (7/81), they clustered in larger or trigonal/supratrigonal defects, supporting the view that defect size and location remain key technical challenges. All failures were successfully re-repaired, consistent with reports that ultimate closure approaches 100% with staged management in specialized centers [28].

Special scenarios were addressed effectively. When the fistula compromised a ureteric orifice, simultaneous ureteric reimplantation achieved complete healing, underscoring the importance of systematic evaluation of ureteral involvement and definitive reconstruction when indicated. Two patients with severely contracted bladders underwent augmentation ileo-cystoplasty at the time of repair, with favorable functional outcomes demonstrating that augmentation, while uncommon, is feasible and effective in selected cases.

All UVF were ultimately cured. Conservative double-J stenting closed 2/3 early-identified cases, a range consistent with published reports where timing and stricture length drive success; definitive extravesical reimplantation yielded 100% closure in our surgically managed UVF, comparable to the ~95-100% success reported across centers [29]. VUF, all post-cesarean, were entirely closed with either laparoscopic or open repair and routine omental interposition, again mirroring the generally favorable prognosis of VUF with standard layered closure. UVVF illustrated the spectrum of complexity: two limited proximal defects closed with layered transvaginal repair plus Martius; two with extensive bladder-neck/urethral destruction required bladder-neck closure and continent catheterizable channels (Mitrofanoff/Monti) to ensure dryness, and it is an accepted last-resort strategy when the continence mechanism is irreparable and consistent with reports of post-repair stress incontinence risk in urethral/proximal fistulas [30].

Postoperative morbidity was low and similar across approaches. There were no Grade IV/V events; most complications were minor (Grade I-II). Seven patients (6.7%) required re-intervention for persistent (Grade III) symptoms, after which continence was achieved. The absence of transfusion across all 104 cases and moderate lengths of stay reflects the safety of modern fistula surgery when core principles of tissue preservation, broad mobilization, tension-free multilayer closure, watertight suture lines, and appropriate drainage are respected [31].

Limitations

Non-randomized, pragmatic selection introduces confounding by indication (with more complex cases in the abdominal groups). Follow-up was limited to three months and may miss late recurrences or functional sequelae (e.g., stress incontinence) [32]. Single-center expertise may limit generalizability.

## Conclusions

Surgical repair of lower genitourinary tract fistulas achieved a 91% short-term success rate, with low complication rates across laparoscopic, open, and transvaginal approaches. While closure rates were comparable, each technique offered distinct advantages. The routine use of interposition flaps and selective adjunctive procedures further strengthened outcomes. These findings emphasize that tailoring the surgical approach to fistula type, size, and complexity enables high success with minimal morbidity, guiding urologists toward evidence-based, individualized management.
